# Supplementary material for: Physiological architecture and evolutionary origins of cellular adaptability
Source: bioRxiv. 2026 Apr 11:2026.04.10.717775. Preprint. [Version 1] doi: 10.64898/2026.04.10.717775 (PMC13081815; doi:10.64898/2026.04.10.717775)
Supplement: Supplement 1 [file NIHPP2026.04.10.717775v1-supplement-1.pdf]

bioRxiv preprint doi: https://doi.org/10.64898/2026.04.10.717775; this version posted April 11, 2026. The copyright holder for this preprint (which was not certified by peer review) is the author/funder, who has granted bioRxiv a license to display the preprint in perpetuity. It is made available under aCC-BY 4.0 International license.

Supplementary Figures

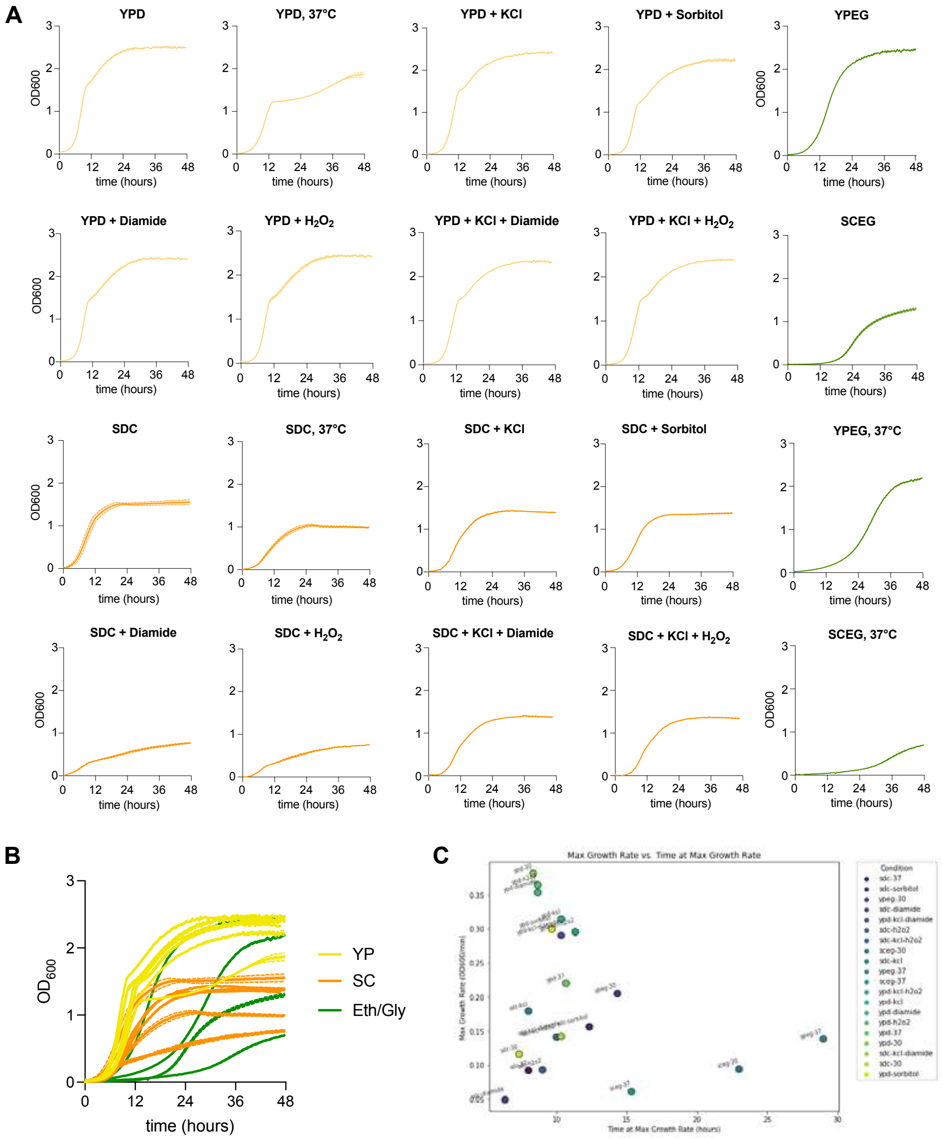

Fig. S1: Growth curves of ancestral strain across environmental conditions. A. OD_{600} versus time for *S. cerevisiae* grown across 20 different complex environments. **B.** OD_{600} versus time for *S. cerevisiae* across environments shown in panel A grouped by whether environment is composed of SDC media (yellow), YPD media (orange), or ethanol/glycerol (green). **C.** Maximum growth rate across all 20 complex environments versus time to reach maximum growth rate of log phase for scRNA-seq collection.

bioRxiv preprint doi: https://doi.org/10.64898/2026.04.10.717775; this version posted April 11, 2026. The copyright holder for this preprint (which was not certified by peer review) is the author/funder, who has granted bioRxiv a license to display the preprint in perpetuity. It is made available under aCC-BY 4.0 International license.

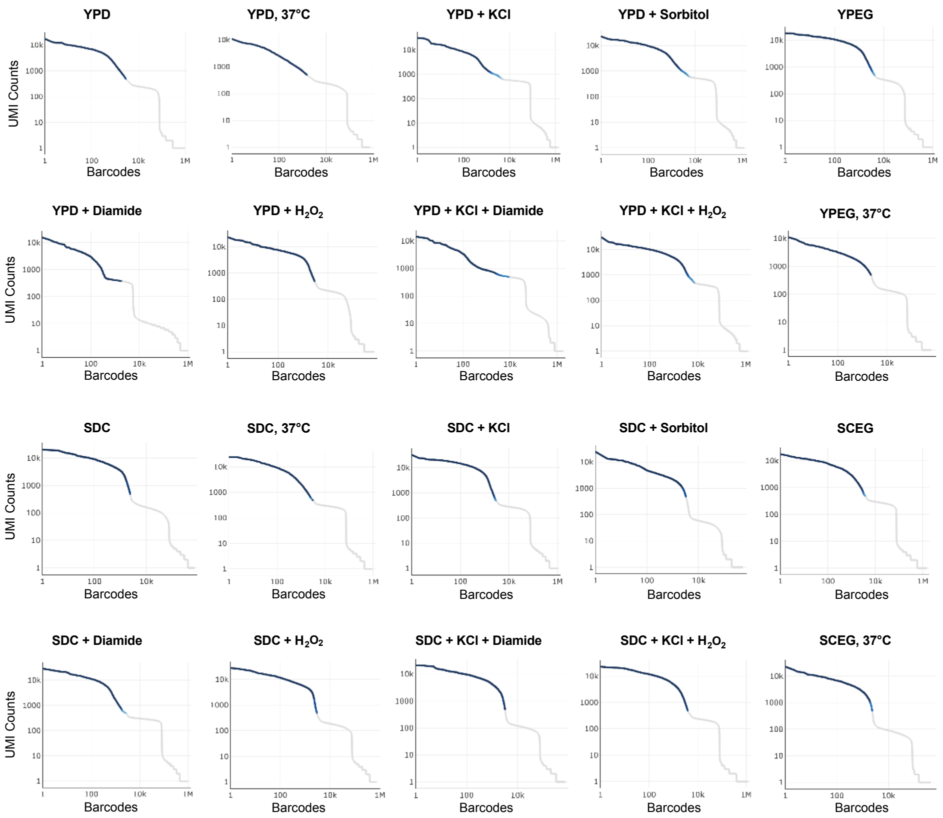

Fig. S2: CellRanger barcode rank plots for ancestral single-cell RNA sequencing conditions. Each panel shows the UMI count per barcode ranked in decreasing order (log–log scale) for one sequencing sample. Dark blue indicates barcodes called as cells; light gray indicates empty droplets. Cell calling was performed using CellRanger v7.1.0.

bioRxiv preprint doi: https://doi.org/10.64898/2026.04.10.717775; this version posted April 11, 2026. The copyright holder for this preprint (which was not certified by peer review) is the author/funder, who has granted bioRxiv a license to display the preprint in perpetuity. It is made available under aCC-BY 4.0 International license.

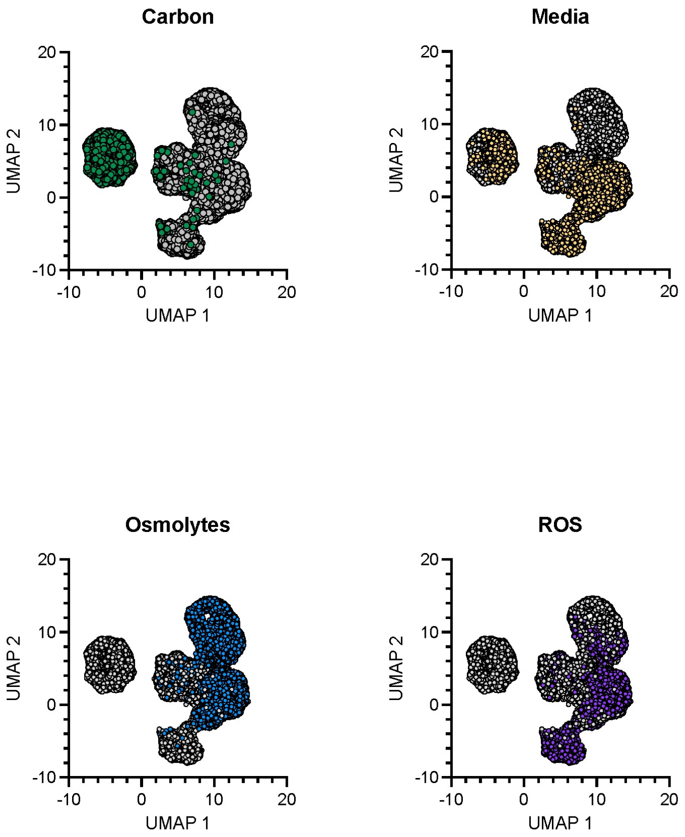

Fig. S3: UMAP colored by individual environmental metadata. UMAP plot of cell populations shown in Fig. 1D colored by various environmental cues.

bioRxiv preprint doi: https://doi.org/10.64898/2026.04.10.717775; this version posted April 11, 2026. The copyright holder for this preprint (which was not certified by peer review) is the author/funder, who has granted bioRxiv a license to display the preprint in perpetuity. It is made available under aCC-BY 4.0 International license.

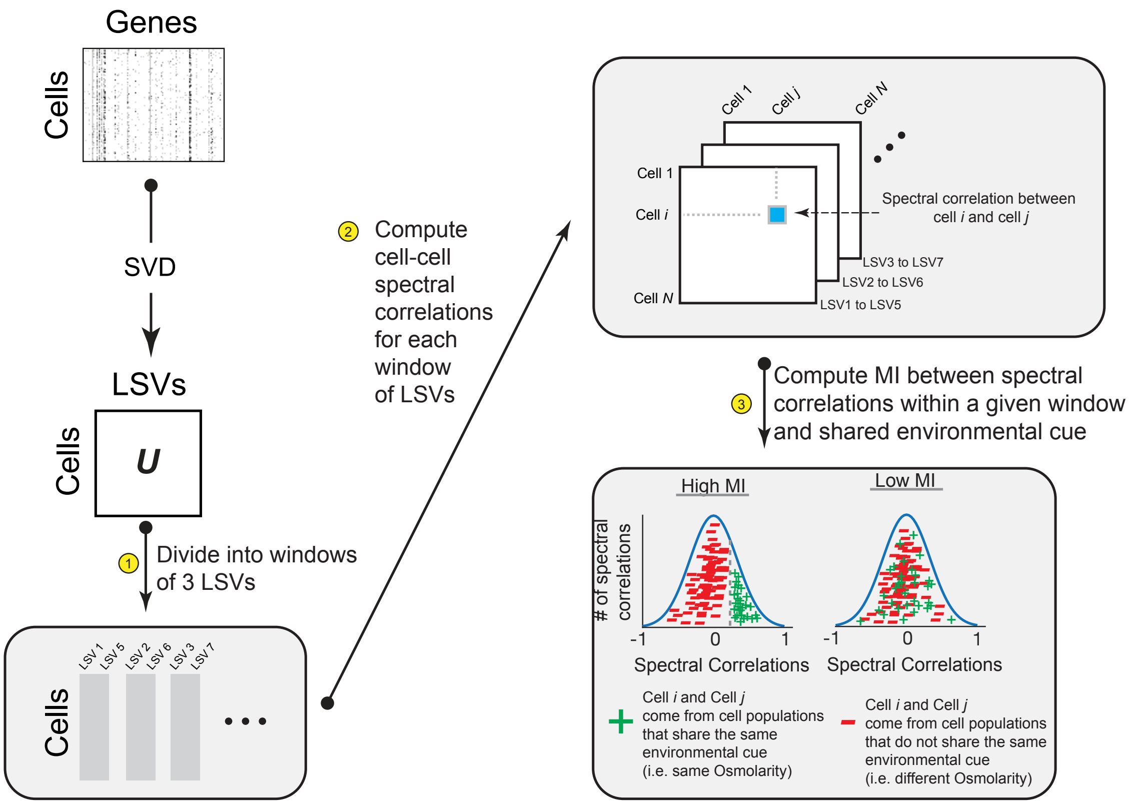

664

Fig. S4: SCALES analysis of environmental information across PCs. Schematic of the Spectral Correlation Analysis of Layered Evolutionary Signals (SCALES) method. A log-normalized gene expression matrix is decomposed by singular value decomposition (SVD). SVD yields a cell embedding matrix U whose columns are the left singular vectors (LSVs), capturing the dominant axes of transcriptional variation across cells. (1) U is partitioned into overlapping windows of 3 consecutive LSVs. (2) Within each window, pairwise spectral correlations are computed between all cells, producing an $N \times N$ correlation matrix. (3) Mutual information (MI) is calculated between the distribution of spectral correlations and the shared environmental label of each cell pair. High MI indicates that the LSV window encodes transcriptional structure that distinguishes environmental conditions; low MI indicates that window carries little discriminatory information.

bioRxiv preprint doi: https://doi.org/10.64898/2026.04.10.717775; this version posted April 11, 2026. The copyright holder for this preprint (which was not certified by peer review) is the author/funder, who has granted bioRxiv a license to display the preprint in perpetuity. It is made available under aCC-BY 4.0 International license.

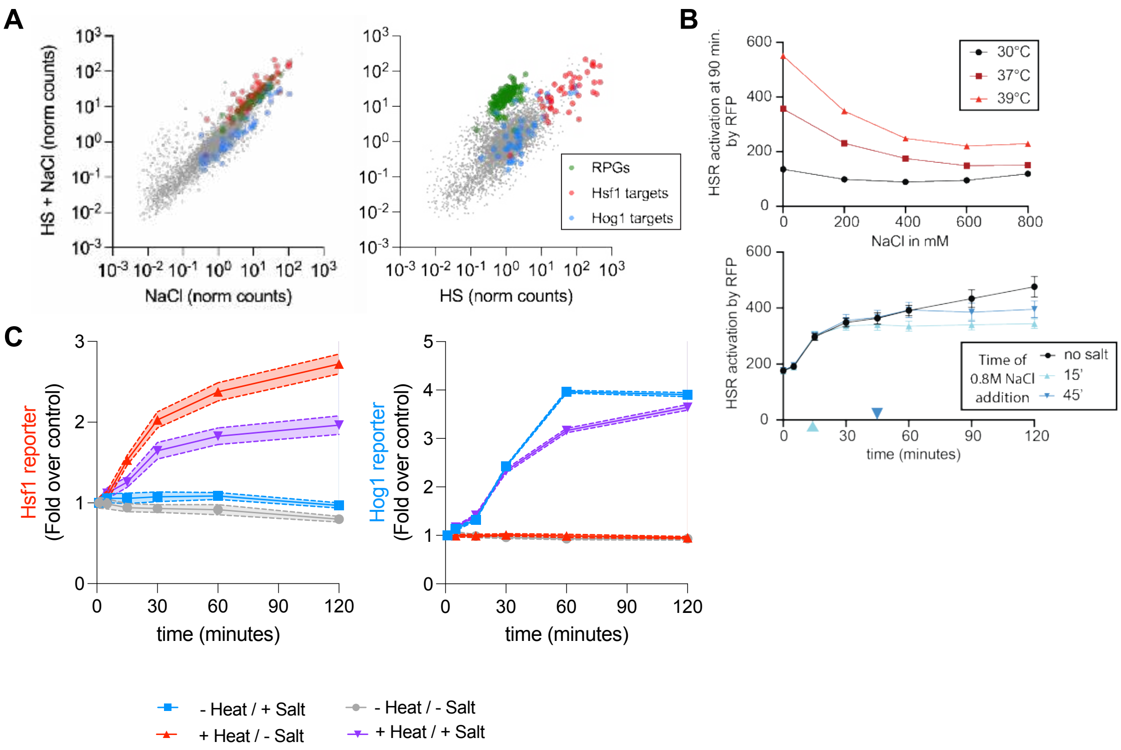

665

Fig. S5: Reporter and transcriptomic validation of environmental epistasis. A. Transcriptome-wide comparison of gene expression in the dual stress condition (HS + NaCl) versus each individual stress. Left: dual stress versus osmotic shock (NaCl) alone; right: dual stress versus heat shock (HS) alone. Normalized read counts are shown on log–log axes with Hsf1 targets (red), Hog1 targets (blue), and ribosomal protein genes (RPGs, green) highlighted. **B.** (top) Dose response matrix of HSE-RFP fluorescent transcriptional reporter across increasing temperature (30°C, 37°C, and 39°C) and osmotic stress (0, 200, 400, 600, 800 mM of NaCl). (bottom) Heat shock time course measurements of HSE-RFP with 800 mM of NaCl introduced at 15 minutes and 45 minutes following heat shock. Each data point represents mean HSE-RFP fluorescence normalized to side scatter (SSC) across 3 biological replicates. **C.** Transcriptional fluorescence reporter measurements of HSE-RFP and pHor2-GFP of cells grown in 2% ethanol/2% glycerol media. Cells are subjected to heat shock (39°C), osmotic shock (0.8 M NaCl), or simultaneous dual stress. Stress response measurements are lower in ethanol/glycerol-grown media compared to glucose media (Fig. 2E).

bioRxiv preprint doi: https://doi.org/10.64898/2026.04.10.717775; this version posted April 11, 2026. The copyright holder for this preprint (which was not certified by peer review) is the author/funder, who has granted bioRxiv a license to display the preprint in perpetuity. It is made available under aCC-BY 4.0 International license.

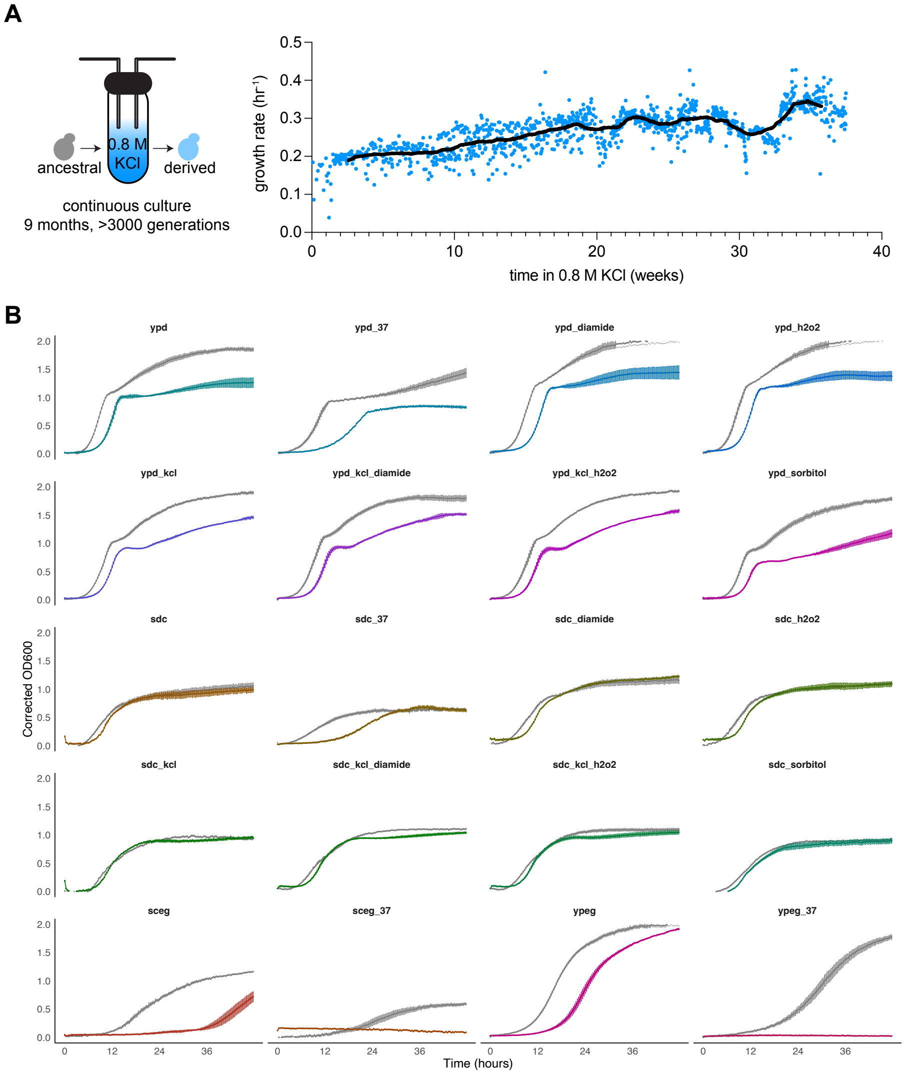

666

Fig. S6: Growth characteristics of the derived strain. A. Growth rate of the evolving strain versus number of weeks of selection under 0.8 M KCl. **B.** OD_{600} growth curve measurements versus time of the derived strain (color), plotted against the ancestral strain (gray). The derived strain exhibited slower growth in most conditions, with the exception of the selection condition. The derived strain is unable to grow at 37°C under ethanol/glycerol media.

bioRxiv preprint doi: https://doi.org/10.64898/2026.04.10.717775; this version posted April 11, 2026. The copyright holder for this preprint (which was not certified by peer review) is the author/funder, who has granted bioRxiv a license to display the preprint in perpetuity. It is made available under aCC-BY 4.0 International license.

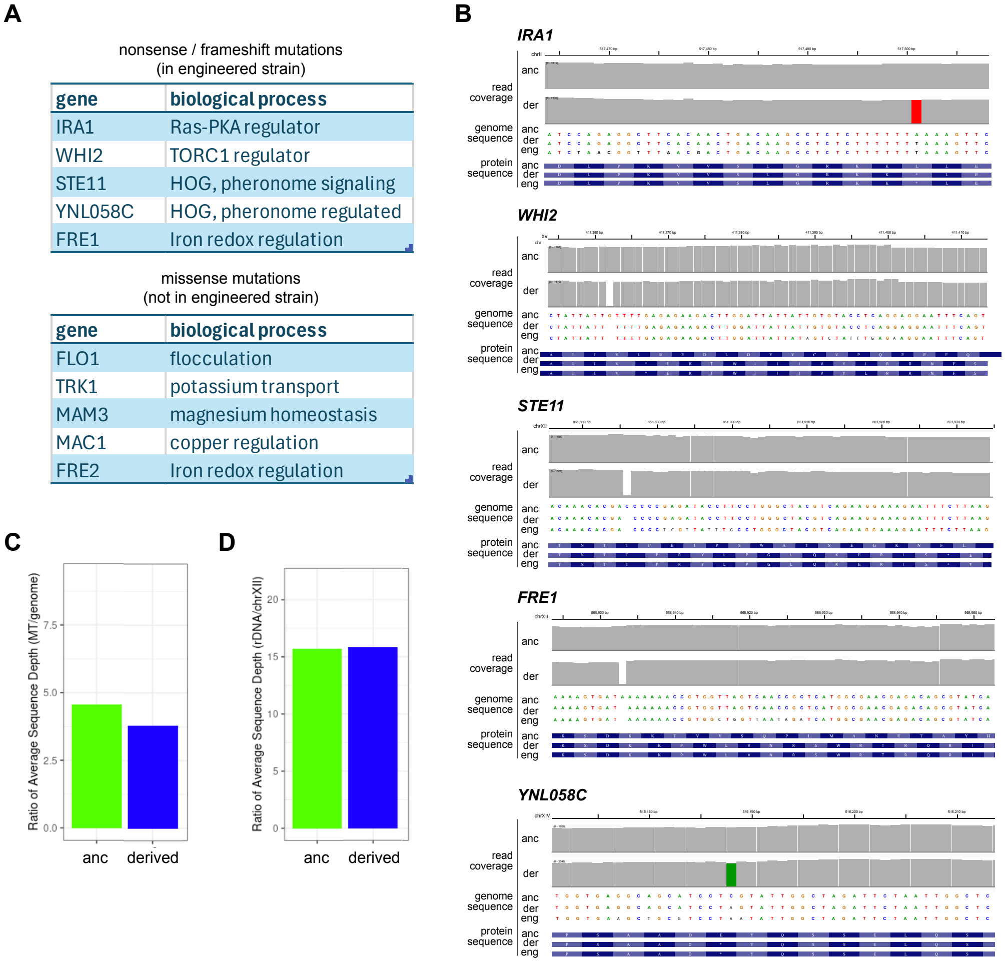

Fig. S7: Genomic characterization of the derived strain. A. Genome-wide view of mutations in the derived strain relative to the ancestral strain, showing nonsense and frameshift mutations (top) and missense mutations (bottom) across all chromosomes. **B.** Read coverage and sequence alignments at the five genes harboring premature termination codons (*IRA1*, *WHI2*, *STE11*, *FRE1*, and *YNL058C*), comparing the parental (P), derived (V0), and CRISPR-engineered (5gm) strains. **C.** Ratio of average sequencing depth for the mitochondrial genome relative to the nuclear genome in ancestral and derived strains. **D.** Ratio of average sequencing depth for the rDNA locus on chromosome XII relative to the rest of the nuclear genome in ancestral and derived strains.

bioRxiv preprint doi: https://doi.org/10.64898/2026.04.10.717775; this version posted April 11, 2026. The copyright holder for this preprint (which was not certified by peer review) is the author/funder, who has granted bioRxiv a license to display the preprint in perpetuity. It is made available under aCC-BY 4.0 International license.

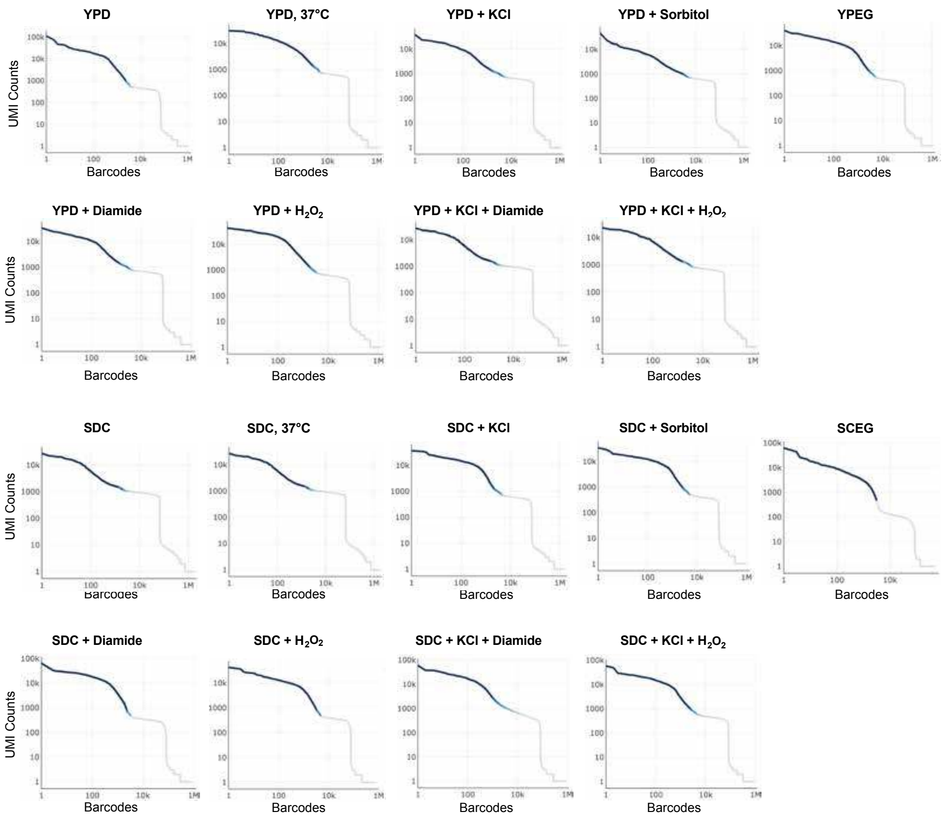

Fig. S8: CellRanger barcode rank plots for derived strain single-cell RNA sequencing conditions. Each panel shows the UMI count per barcode ranked in decreasing order (log–log scale) for one sequencing sample. Dark blue indicates barcodes called as cells; light gray indicates empty droplets. Cell calling was performed using CellRanger v7.1.0.

bioRxiv preprint doi: https://doi.org/10.64898/2026.04.10.717775; this version posted April 11, 2026. The copyright holder for this preprint (which was not certified by peer review) is the author/funder, who has granted bioRxiv a license to display the preprint in perpetuity. It is made available under aCC-BY 4.0 International license.

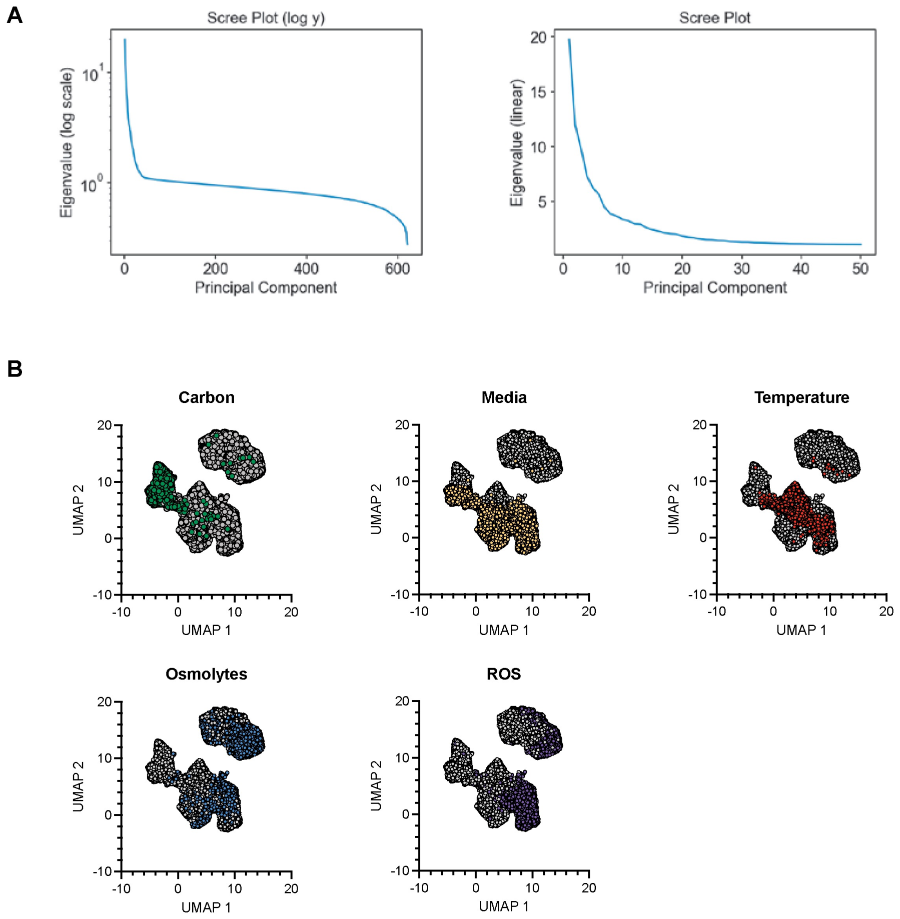

669

Fig. S9: Scree plots and UMAP analysis of the derived strain scRNA-seq data. A. Fraction of total variance explained by each left singular vector (LSV) following SVD of the log-normalized gene expression matrix of the derived strain. **B.** UMAP plot of derived strain cell populations colored by various environmental cues.

bioRxiv preprint doi: https://doi.org/10.64898/2026.04.10.717775; this version posted April 11, 2026. The copyright holder for this preprint (which was not certified by peer review) is the author/funder, who has granted bioRxiv a license to display the preprint in perpetuity. It is made available under aCC-BY 4.0 International license.

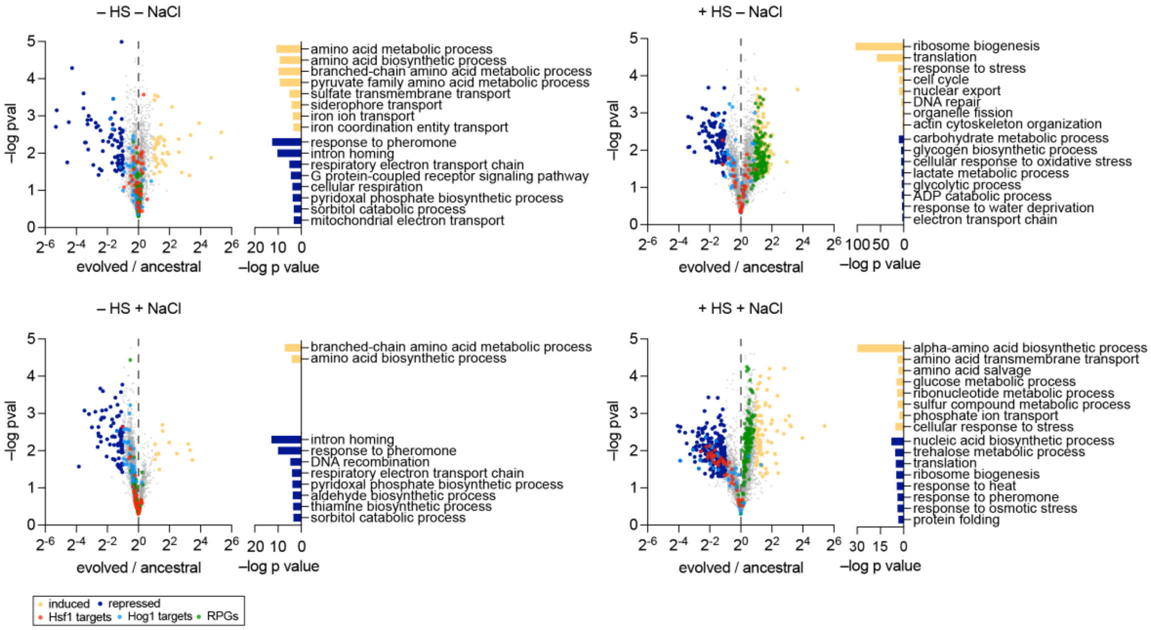

Fig. S10: Differential gene expression and GO term enrichment in derived cells across stress conditions. Differential gene expression between the derived and ancestral strains across four conditions: unstressed (−HS −NaCl), heat shock (+HS −NaCl), osmotic shock (−HS +NaCl), and dual stress (+HS +NaCl). (Left on each panel) Volcano plots showing bulk RNA-seq differential expression between derived and ancestral cells. Colored points indicate Hsf1 target genes (red), Hog1 target genes (green), and ribosomal protein genes (RPGs, blue). (Right on each panel) GO biological process enrichment of genes induced (orange) or repressed (blue) in evolved cells.

bioRxiv preprint doi: https://doi.org/10.64898/2026.04.10.717775; this version posted April 11, 2026. The copyright holder for this preprint (which was not certified by peer review) is the author/funder, who has granted bioRxiv a license to display the preprint in perpetuity. It is made available under aCC-BY 4.0 International license.

Supplementary Tables

Table S1: QC metrics of single-cell sequencing runs. Summary statistics from CellRanger v7.1.0 for all ancestral (anc) and evolved (evo) scRNA-seq samples.

Condition	Reads	Valid BC (%)	Sat. (%)	Q30 (%)	Map Genome (%)	Map Txome (%)	Cells	Med. Genes	Med. UMI
Ancestral strain									
SCEG-30	160M	97.3	66.0	95.6	75.4	53.5	3,353	902	1,688
SCEG-37	160M	96.7	80.9	96.0	64.1	39.9	2,212	1,172	2,416
SDC	145M	97.4	79.5	94.8	78.8	60.1	2,313	1,219	3,119
SDC-37	172M	97.3	70.0	95.5	75.4	52.4	2,767	902	1,429
SDC-diamide	146M	97.4	59.4	95.7	74.4	51.7	2,056	844	1,620
SDC-H_2O_2	158M	97.4	69.4	95.2	76.3	51.9	2,813	1,362	4,314
SDC-KCl	169M	98.0	72.2	94.8	85.2	67.3	2,756	1,348	3,271
SDC-KCl-dia	147M	97.7	77.5	95.4	82.9	59.4	3,191	1,303	2,958
SDC-KCl-H_2O_2	175M	97.4	69.7	95.0	79.5	57.7	3,844	1,206	2,644
SDC-sorbitol	132M	97.6	85.4	95.7	78.5	54.3	3,123	939	1,701
YPD	149M	97.1	73.6	95.8	75.9	57.7	2,844	744	1,180
YPD-37	142M	96.5	74.7	93.3	68.5	46.7	1,534	522	828
YPD-diamide	140M	97.7	93.5	95.4	82.2	62.2	1,896	311	416
YPD-H_2O_2	164M	97.1	79.1	95.7	76.3	57.8	2,862	1,217	2,506
YPD-KCl	159M	97.9	46.1	95.8	81.8	59.5	2,846	653	1,262
YPD-KCl-dia	142M	97.3	61.6	94.6	79.6	56.6	6,343	358	590
YPD-KCl-H_2O_2	163M	97.8	54.0	95.7	85.4	60.7	5,440	960	1,968
YPD-sorbitol	130M	97.6	41.0	95.6	81.7	60.3	4,236	680	1,286
YPEG-30	160M	97.7	67.4	95.1	81.0	58.3	3,659	1,078	2,178
YPEG-37	115M	96.5	75.6	95.3	65.7	40.9	2,260	675	1,113
Derived strain									
SCEG-30	138M	95.8	75.8	96.8	73.1	52.6	3,081	1,008	2,092
SDC	159M	97.7	61.1	96.9	84.0	61.8	3,114	1,106	2,368
SDC-37	166M	98.2	55.1	97.0	87.3	66.4	3,623	1,181	2,667
SDC-diamide	168M	96.9	54.3	97.0	80.6	50.4	2,663	1,108	2,578
SDC-H_2O_2	158M	97.9	58.2	97.0	88.6	61.4	4,040	1,061	2,030
SDC-KCl	177M	98.1	48.0	97.0	89.5	59.8	3,571	783	1,607
SDC-KCl-dia	164M	98.2	52.8	97.0	89.4	64.8	3,421	670	1,312
SDC-KCl-H_2O_2	141M	97.3	49.6	96.8	82.1	65.1	3,573	708	1,379
SDC-sorbitol	149M	97.0	53.0	97.0	79.4	57.2	3,792	832	1,426
YPD	137M	96.4	50.1	96.9	78.4	61.9	2,997	1,011	1,924
YPD-37	179M	98.2	45.4	97.0	88.3	59.3	4,127	863	1,705
YPD-diamide	141M	98.2	43.7	96.6	87.2	69.8	2,842	670	1,367
YPD-H_2O_2	157M	97.4	47.4	97.0	81.8	62.3	3,090	718	1,610
YPD-KCl	140M	97.2	38.2	97.0	82.7	66.8	4,165	570	1,084
YPD-KCl-dia	167M	98.4	44.8	97.1	89.1	70.7	2,747	594	1,508
YPD-KCl-H_2O_2	147M	97.4	42.5	96.8	82.2	65.8	3,254	598	1,322
YPD-sorbitol	118M	96.7	36.9	96.9	78.6	62.1	4,773	637	1,084
YPEG-30	171M	98.0	61.4	97.0	88.5	66.9	3,779	799	1,518

bioRxiv preprint doi: https://doi.org/10.64898/2026.04.10.717775; this version posted April 11, 2026. The copyright holder for this preprint (which was not certified by peer review) is the author/funder, who has granted bioRxiv a license to display the preprint in perpetuity. It is made available under aCC-BY 4.0 International license.

Table S2: Top 10 gene loadings per principal component (ancestral strain). For each of the first five principal components from SVD of the ancestral scRNA-seq dataset, the 10 genes with the largest absolute loadings are shown with their loading values. Positive and negative loadings indicate opposing directions of transcriptional variation along each PC.

PC1			PC2			PC3			PC4			PC5		
**#**	**Gene**	**Loading**	**#**	**Gene**	**Loading**	**#**	**Gene**	**Loading**	**#**	**Gene**	**Loading**	**#**	**Gene**	**Loading**
1	*TDH3*	−0.116	1	*INO1*	0.104	1	*INO1*	0.148	1	*OLI1*	−0.214	1	*PDC5*	0.137
2	*CCW12*	−0.108	2	*RPL9A*	−0.091	2	*HSP82*	−0.134	2	*21S_RRNA*	−0.197	2	*ENO2*	0.136
3	*PDC1*	−0.103	3	*RPS9B*	−0.081	3	*ZRT1*	0.131	3	*15S_RRNA*	−0.160	3	*TDH3*	0.122
4	*ENO2*	−0.101	4	*GAP1*	0.081	4	*CYC1*	−0.114	4	*CWP2*	−0.131	4	*21S_RRNA*	0.120
5	*FBA1*	−0.098	5	*RPS22A*	−0.080	5	*MET6*	0.114	5	*FIT3*	−0.122	5	*RIB4*	0.114
6	*CDC19*	−0.090	6	*MET6*	0.079	6	*PDC5*	0.112	6	*CCW12*	−0.119	6	*GPM1*	0.112
7	*CWP2*	−0.085	7	*ARG4*	0.079	7	*HSP60*	−0.112	7	*FIT2*	−0.114	7	*PDC1*	0.110
8	*21S_RRNA*	−0.083	8	*RPS31*	−0.076	8	*SHM2*	0.111	8	*INO1*	−0.096	8	*CDC19*	0.107
9	*GPM1*	−0.083	9	*FIT2*	0.075	9	*HSP10*	−0.103	9	*TDH3*	−0.090	9	*HTB1*	0.105
10	*TPI1*	−0.082	10	*ZRT1*	0.075	10	*SSA1*	−0.097	10	*AI1*	−0.088	10	*FBA1*	0.101

bioRxiv preprint doi: https://doi.org/10.64898/2026.04.10.717775; this version posted April 11, 2026. The copyright holder for this preprint (which was not certified by peer review) is the author/funder, who has granted bioRxiv a license to display the preprint in perpetuity. It is made available under aCC-BY 4.0 International license.

Table S3: Genomic variants in the derived strain (HIGH and MODERATE impact). Variants were identified by whole-genome sequencing and annotated using SnpEff relative to the *S. cerevisiae* R64-4-1 reference genome. Only variants with HIGH or MODERATE (MOD) predicted impact are shown. HIGH-impact variants (premature stop codons and frameshifts) are shown in bold.

ID	Chr	Position	Alt	Annotation	Impact	Gene	HGVS.c	HGVS.p
**V0-16**	**chrII**	**517501**	**T**	**stop_gained**	**HIGH**	***IRA1***	**c.9128T>A**	**p.Leu3043***
**V0-67**	**chrXII**	**568902**	**T**	**frameshift**	**HIGH**	***FRE1***	**c.343delA**	**p.Thr115fs**
**V0-71**	**chrXII**	**851885**	**A**	**frameshift**	**HIGH**	***STE11***	**c.2025delC**	**p.Glu676fs**
**V0-83**	**chrXIV**	**516188**	**A**	**stop_gained**	**HIGH**	***YNL058C***	**c.526G>T**	**p.Glu176***
**V0-90**	**chrXV**	**411361**	**T**	**frameshift**	**HIGH**	***WHI2***	**c.493delG**	**p.Val165fs**
**V0-92**	**chrXVI**	**810734**	**A**	**frameshift**	**HIGH**	**tN(GUU)P**	**c.62delC**	**p.Pro21fs**
V0-13	chrII	429	G	missense	MOD	*YBL113C*	c.2230G>C	p.Val744Leu
V0-14	chrII	137262	T	missense	MOD	*ECM13*	c.575C>T	p.Ala192Val
V0-17	chrII	675115	C	missense	MOD	*MCX1*	c.20A>G	p.Gln7Arg
V0-26	chrIV	397109	A	missense	MOD	*DBP10*	c.2893G>A	p.Glu965Lys
V0-27	chrIV	608805	T	missense	MOD	*PDC2*	c.1277C>A	p.Ala426Glu
V0-42	chrV	329736	C	missense	MOD	*ILV1*	c.1260A>C	p.Glu420Asp
V0-43	chrVII	39951	C	missense	MOD	*GUS1*	c.929G>C	p.Gly310Ala
V0-59	chrX	175779	A	missense	MOD	*TRK1*	c.1528G>T	p.Asp510Tyr
V0-61	chrXI	11174	T	missense	MOD	*FRE2*	c.53C>A	p.Ala18Glu
V0-62	chrXI	78356	A	missense	MOD	*MST1*	c.291G>T	p.Lys97Asn
V0-63	chrXI	516811	C	missense	MOD	*GAP1*	c.1749A>C	p.Glu583Asp
V0-77	chrXIII	318217	A	missense	MOD	*MAC1*	c.202G>T	p.Asp68Tyr
V0-78	chrXIII	426039	A	missense	MOD	*SEC14*	c.895G>A	p.Glu299Lys
V0-89	chrXV	216004	T	missense	MOD	*MAM3*	c.134C>A	p.Ser45Tyr

bioRxiv preprint doi: https://doi.org/10.64898/2026.04.10.717775; this version posted April 11, 2026. The copyright holder for this preprint (which was not certified by peer review) is the author/funder, who has granted bioRxiv a license to display the preprint in perpetuity. It is made available under aCC-BY 4.0 International license.

Table S4: Top 10 gene loadings per principal component (derived strain). For each of the first five principal components from SVD of the derived scRNA-seq dataset, the 10 genes with the largest absolute loadings are shown with their loading values. Positive and negative loadings indicate opposing directions of transcriptional variation along each PC.

PC1			PC2			PC3			PC4			PC5		
**#**	**Gene**	**Loading**	**#**	**Gene**	**Loading**	**#**	**Gene**	**Loading**	**#**	**Gene**	**Loading**	**#**	**Gene**	**Loading**
1	*TDH3*	−0.116	1	*INO1*	0.104	1	*INO1*	0.148	1	*OLI1*	−0.214	1	*PDC5*	0.137
2	*CCW12*	−0.108	2	*RPL9A*	−0.091	2	*HSP82*	−0.134	2	*21S_RRNA*	−0.197	2	*ENO2*	0.136
3	*PDC1*	−0.103	3	*RPS9B*	−0.081	3	*ZRT1*	0.131	3	*15S_RRNA*	−0.160	3	*TDH3*	0.122
4	*ENO2*	−0.101	4	*GAP1*	0.081	4	*CYC1*	−0.114	4	*CWP2*	−0.131	4	*21S_RRNA*	0.120
5	*FBA1*	−0.098	5	*RPS22A*	−0.080	5	*MET6*	0.114	5	*FIT3*	−0.122	5	*RIB4*	0.114
6	*CDC19*	−0.090	6	*MET6*	0.079	6	*PDC5*	0.112	6	*CCW12*	−0.119	6	*GPM1*	0.112
7	*CWP2*	−0.085	7	*ARG4*	0.079	7	*HSP60*	−0.112	7	*FIT2*	−0.114	7	*PDC1*	0.110
8	*21S_RRNA*	−0.083	8	*RPS31*	−0.076	8	*SHM2*	0.111	8	*INO1*	−0.096	8	*CDC19*	0.107
9	*GPM1*	−0.083	9	*FIT2*	0.075	9	*HSP10*	−0.103	9	*TDH3*	−0.090	9	*HTB1*	0.105
10	*TPI1*	−0.082	10	*ZRT1*	0.075	10	*SSA1*	−0.097	10	*AI1*	−0.088	10	*FBA1*	0.101

bioRxiv preprint doi: https://doi.org/10.64898/2026.04.10.717775; this version posted April 11, 2026. The copyright holder for this preprint (which was not certified by peer review) is the author/funder, who has granted bioRxiv a license to display the preprint in perpetuity. It is made available under aCC-BY 4.0 International license.

Table S5: Yeast strains used in this study. All strains are in the W303 background (*MAT**a***; *leu2-3,112; trp1-1; can1-100; ura3-1; ade2-1; his3-11,15*).

Strain	Genotype	Bg	Description	Figures
DPY144	4×HSE-Venus::LEU2	W303	WT HSE reporter	S1, 2D, 2F, 3C, 3E, 3F, S3D, 5B, S5
DPY1284	*tpk1/2/3-as*; *TOR1-1*(S1972I); *fpr1*Δ::NAT; TEF2pr-mKate-URA3-4×HSEve-EmGFP; RPL13A-2×FKBP12::TRP; SIS1-FRB::HIS	W303	Sis1 anchor-away	1E, 2B
DPY1368	HSEv2-Venus::LEU2; *ssa1*Δ::HYG; *ssa2*Δ::KAN; TEF1pr-SSA2::HIS; *ssa4*Δ::NAT; TEF1pr-SSA2::URA; *ssa3*Δ::BLE	W303	ΔFBL Hsp70	3C, 3E, 3F, S3C, 5A, 5B
DPY1600	4×HSE-Venus::LEU2; Hsp82-ERVB1-mScarlet::KAN	W303	Hsp82-P2A-mScarlet	S1, S3C, 5A
DPY1615	4×HSE-Venus::LEU2; Sis1-ERVB1-mScarlet::KAN	W303	SIS1pr-SIS1-P2A-mScarlet	S3A, S3B, S3D
DPY1661	4×HSE-Venus::LEU2; Sup35pr-Sis1-ERVB1-mScarlet::KAN	W303	Sup35pr-SIS1-P2A-mScarlet	S3A, S3B, S3D
DPY1701	4×HSE-Venus::LEU2; Sup35pr-SIS1	W303	Sup35pr-SIS1	S3C, S3D
DPY1761	4×HSE-Venus::LEU2; Sup35pr-SIS1; Sup35pr-Sis1::TRP	W303	2× Sup35pr-SIS1	3C, 3E, 3F, S3C, S3D, 5B, 5A, S5
DPY1846	Sis1-mVenus::HIS; Nsr1-mScarletI::KAN; *ssa1*pr-HALO-*ssa1*ORF::LEU	W303	Ssa1-HaloTag imaging	4B, 4D
DPY1930	HALO-Sis1; Nsr1-mScarletI::KAN	W303	Sis1-HaloTag imaging	4B, 4C
DPY1990	Hsf1-mVenus::HIS; *ssa1*pr-HALO-*ssa1*ORF::LEU	W303	Ssa1-HaloTag; Hsf1-mVenus	4E, 4F
DPY1991	4×HSE-mVenus::HIS; Pab1-mKate::URA3	W303	Pab1-mKate	5C
DPY1992	4×HSE-Venus::LEU2; Sup35pr-SIS1; Sup35pr-Sis1::TRP; Pab1-mKate::URA3	W303	Pab1-mKate; 2× Sup35pr-SIS1	5C
DPY2001	HALO-Sis1 (N-terminal CRISPR); Hsf1-mVenus::HIS	W303	HALO-Sis1; Hsf1-mVenus	4E, 4F
Additional strains				
—	LEU2::HSE-mApple; TRP1::pHOR2-GFP	W303	HSR/HOG dual reporter	2
—	Hog1-[FP]; Hsf1-[FP] (endogenous tagged)	W303	Hog1/Hsf1 nuclear localization	3B, 3C
—	Sis1-Venus::HIS3; Nsr1-mScarletI::KAN; Rpl26a-HaloTag	W303	oRP/Sis1 co-localization	3E, 3F
Derived (V0)	W303 + IRA1(L3043*); STE11(E676fs); WHI2(V165fs); FRE1(T115fs); YNL058C(E176*); petite	W303[†]	Derived strain (>3000 gen. KCl)	4–6, S6–S8
5gm (CRISPR)	W303 + IRA1(L3043*); STE11(E676fs); WHI2(V165fs); FRE1(T115fs); YNL058C(E176*)	W303	CRISPR reconstruction	4B, S7B
DPY731	TDH3pr-mKate2	W303	mKate2 reference strain	4B

bioRxiv preprint doi: https://doi.org/10.64898/2026.04.10.717775; this version posted April 11, 2026. The copyright holder for this preprint (which was not certified by peer review) is the author/funder, who has granted bioRxiv a license to display the preprint in perpetuity. It is made available under aCC-BY 4.0 International license.

Table S6: Environmental conditions. The 20 environments used for transcriptional profiling and growth assays. YP = yeast extract + peptone (rich); SC = synthetic complete (defined). All conditions at 30°C unless otherwise noted.

#	Label	Media	Carbon source	Temp.	Osmolyte	ROS agent	Notes
1	YPD-30	YP	2% glucose	30°C	—	—	Reference
2	YPD-37	YP	2% glucose	37°C	—	—	Heat stress
3	YPD-KCl	YP	2% glucose	30°C	500 mM KCl	—	Ionic osmotic
4	YPD-sorbitol	YP	2% glucose	30°C	500 mM sorbitol	—	Non-ionic osmotic
5	YPD-diamide	YP	2% glucose	30°C	—	50 µM diamide	Thiol oxidant
6	YPD-H_2O_2	YP	2% glucose	30°C	—	50 µM H_2O_2	ROS
7	YPD-KCl-diamide	YP	2% glucose	30°C	500 mM KCl	50 µM diamide	Osmotic + ROS
8	YPD-KCl-H_2O_2	YP	2% glucose	30°C	500 mM KCl	50 µM H_2O_2	Osmotic + ROS
9	SDC-30	SC	2% glucose	30°C	—	—	Defined media
10	SDC-37	SC	2% glucose	37°C	—	—	Defined + heat
11	SDC-KCl	SC	2% glucose	30°C	500 mM KCl	—	Evolution condition*
12	SDC-sorbitol	SC	2% glucose	30°C	500 mM sorbitol	—	Defined + non-ionic
13	SDC-diamide	SC	2% glucose	30°C	—	50 µM diamide	Defined + thiol oxidant
14	SDC-H_2O_2	SC	2% glucose	30°C	—	50 µM H_2O_2	Defined + ROS
15	SDC-KCl-diamide	SC	2% glucose	30°C	500 mM KCl	50 µM diamide	Defined + osmotic/ROS
16	SDC-KCl-H_2O_2	SC	2% glucose	30°C	500 mM KCl	50 µM H_2O_2	Defined + osmotic/ROS
17	YPEG-30	YP	2% EtOH/2% glycerol	30°C	—	—	Non-fermentable
18	YPEG-37	YP	2% EtOH/2% glycerol	37°C	—	—	Non-ferm. + heat
19	SCEG-30	SC	2% EtOH/2% glycerol	30°C	—	—	Defined + non-ferm.
20	SCEG-37	SC	2% EtOH/2% glycerol	37°C	—	—	Defined + non-ferm. + heat†

*eVOLVER evolution used 0.8 M KCl in SDC. †Derived strain failed to grow in this condition.

bioRxiv preprint doi: https://doi.org/10.64898/2026.04.10.717775; this version posted April 11, 2026. The copyright holder for this preprint (which was not certified by peer review) is the author/funder, who has granted bioRxiv a license to display the preprint in perpetuity. It is made available under aCC-BY 4.0 International license.

Table S7: CRISPR guide RNA and repair template sequences. Guide RNAs were designed near each variant site and scored using the system of Joung et al. [2017]. Repair templates introduce the derived-strain mutation along with synonymous PAM-site mutations (lowercase) to prevent repeated Cas9 cleavage. Engineering was performed using the pCRCT system [Bao et al., 2015] (Addgene #60621).

Target gene / Mutation	gRNA (5′→3′)	Repair template (5′→3′)
IRA1<br>p.Leu3043* (stop gained)<br>chrII:517501 A→T	CTTGTCAGTTGT<br>GAAGCCTC	AACTTTTCTATATGTTCCTGATCAAAAATATC<br>tAacGGtTTaACgACTGACAAGCCTCTCTTTTTT<br>tAAAGTTCGACCATTTCATCATTAAATTCCTCTA
WHI2<br>p.Val165fs (frameshift)<br>chrXV:411361 TG→T	GGATTATTATTG<br>TGTACCTC	GGATATTTTACATCAAAAGCCCGCTATTATTTTTT<br>GAGAGAAGACTTGGATTATTATaGTcTAttTgAGa<br>AGGAATTTCAGTTTGATTCCACTAATGAAG
STE11<br>p.Glu676fs (frameshift)<br>chrXII:851885 AC→A	AGCCCAGGAAGG<br>TATCTCGG	TTTCTCAAATGCAAGCGATCTTCAAAATAGGCAC<br>AAACACGACCCCtcGtTAttTgCCTGGGCTACGTC<br>AGAAGGAAAGAATTTCTTAAGAAAGGCATTT
FRE1<br>p.Thr115fs (frameshift)<br>chrXII:568902 TA→T	CATGAGCGGTTG<br>ACTAACCA	ATGCGTCAAATTATTTGAGAGCACCTGAGAAAAGT<br>GATAAAAAAACCGTGGcTgGTtAAtaGaTCATGGCG<br>AACGAGACAGCGTATCATTATTATTTATGAG
YNL058C<br>p.Glu176* (stop gained)<br>chrXIV:516188 C→A	GTGAGGCAGCAT<br>CCTCGTAT	CTGTTGTCTGTGAACGGATCCTGAATTGGGTTGA<br>ATGGTGAaGCtGCgTCCTaaTATTGaCTAGATTCT<br>AATTGGCTATAGAATCCCAATCATCCTGCA
